# Increased aqueous autotaxin and lysophosphatidic acid levels are potential prognostic factors after trabeculectomy in different types of glaucoma

**DOI:** 10.1038/s41598-018-29649-3

**Published:** 2018-07-27

**Authors:** Nozomi Igarashi, Megumi Honjo, Makoto Kurano, Yutaka Yatomi, Koji Igarashi, Kuniyuki Kano, Junken Aoki, Makoto Aihara

**Affiliations:** 1 Department of Ophthalmology, Graduate School of Medicine, The University of Tokyo, Tokyo, Japan; 2 Department of Clinical Laboratory Medicine, Graduate School of Medicine, The University of Tokyo, Tokyo, Japan; 3 CREST, Japan Science and Technology Corporation (JST), Saitama, Japan; 4 Department of Clinical Laboratory, The University of Tokyo Hospital, Tokyo, Japan; 5 Bioscience Division, Reagent Development Department, AIA Research Group, TOSOH Corporation, Kanagawa, Japan; 6 Laboratory of Molecular and Cellular Biochemistry, Graduate School of Pharmaceutical Sciences, Tohoku University, Miyagi, Japan

## Abstract

We explored the potential relevance of aqueous lysophosphatidic acid (LPA) and autotaxin (ATX) levels on postoperative outcomes of trabeculectomy, and the effects of ATX on fibrotic response in cultured human conjunctiva fibroblast (HCF) cells. We enrolled 70 glaucomatous eyes which underwent trabeculectomy, and quantified aqueous LPA and ATX. Those eyes were followed up for 12 months, and postoperative filtering blebs were evaluated using anterior segment optical coherence tomography. Also, the ATX-induced fibrotic changes in HCFs and the effects of an ATX inhibitor were assessed. Measured aqueous ATX and LPA levels were significantly different between glaucoma subtypes. In multivariate analyses, aqueous ATX levels were significantly correlated with the presence of needlings at 1, 3, 6 and 12 months after surgery. Exfoliative glaucoma, whose ATX level was significantly high, showed significantly increased numbers of needlings and a lower cumulative success rate without needlings. An *in vitro* study showed that fibrotic changes were upregulated by ATX treatment in HCFs, which was significantly suppressed by an ATX inhibitor. We presently demonstrate that aqueous ATX may be a prognostic factor affecting the fibrotic response in HCFs and bleb formation, and inhibition of ATX could be a therapeutic target after trabeculectomy.

## Introduction

Glaucoma is the second leading cause of blindness worldwide, characterized by aberrant increases in intraocular pressure (IOP) that can damage the optic nerve^1–3^. Reducing IOP is the only effective therapy to prevent visual impairment and blindness, in both hypertensive and normotensive individuals. Trabeculectomy is used most commonly to lower the IOP in glaucoma^4,5^. Despite advances in surgical techniques and postoperative care, excessive scarring and tissue fibrosis resulting from increased human conjunctival fibroblast (HCF) proliferation and extracellular matrix (ECM) deposition of the subconjunctival tissue, and scleral flaps remain the major impediments to impaired filtering bleb formation and reduction of IOP^6,7^. The usefulness of functional filtration bleb formation after trabeculectomy using anterior segment optical coherence tomography (AS-OCT) has recently been reported^8,9^, which determines if blebs need additional treatments, including needling or a reoperation to achieve more successful outcomes^10^.

Various liquid mediators including transforming growth factor-beta (TGF-β), vascular endothelial growth factor (VEGF), connective tissue growth factor (CTGF), monocyte chemotactic protein-1 (MCP-1) and members of the matrix metalloproteinase (MMP) family are involved in ECM production and HCF fibrosis, and the modulation of these factors has been used as a novel strategy for controlling scarring after trabeculectomy^11–16^. We previously reported that lysophosphatidic acid (LPA) stimulated HCFs, resulting in α-smooth muscle actin (SMA) overexpression and fibrosis^17^. LPA has been recognized as a major bioactive lipid mediator influencing fibrosis, and is produced under various conditions in cells and biological fluids, mainly from lysophosphatidylcholine (LPC), predominantly by the generating enzyme, autotaxin (ATX). ATX is a glycoprotein involved in various physiological processes such as fibrosis and cancer survival^18–21^. Human trabecular meshwork (TM) cells express three isoforms (α, β, γ) of ATX, and ATX has been reported in the aqueous humor (AH)^22^. Primary open-angle glaucoma (POAG) patients have higher ATX activity, which is used to convert LPC into LPA^22^. Recently, we reported that aqueous ATX and LPA concentrations were significantly correlated with IOP and higher in secondary glaucoma (SOAG)^23^. To the best of our knowledge, there have been no reports comparing the levels of ATX and LPA in different glaucoma subtypes, and their involvement in fibrosis after trabeculectomy. In this study, we evaluated the association between aqueous levels of ATX/LPA and bleb morphology using AS-OCT after trabeculectomy in different glaucoma subtypes and characterized the *in vitro* effects of ATX and an ATX inhibitor in the fibrotic response of HCFs.

## Results

### Comparison of IOP and ATX levels in the AH between glaucoma subtypes

**Figure 1:**
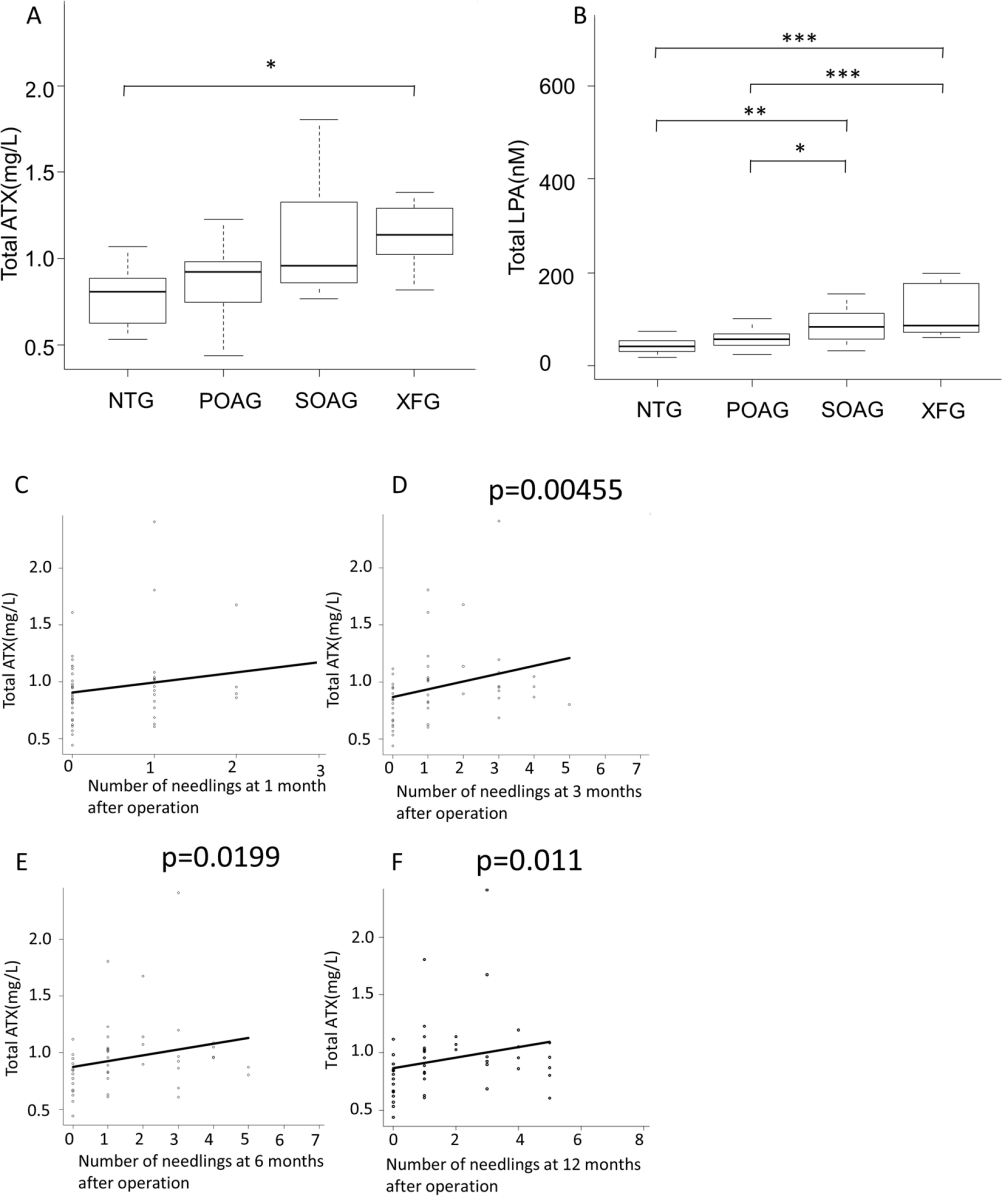
Relationships between aqueous ATX, LPA levels and glaucoma subtypes and the total sum of needlings. Relationships between aqueous ATX **(A)** and LPA **(B)** levels and glaucoma subtypes. **(A)** The ATX level measured by immunoenzymetric assay was significantly higher in the XFG group compared to other groups (P < 0.05). **(B)** Total LPA measured using liquid chromatography-tandem mass spectrometry was significantly higher in the XFG and SOAG groups compared to normal-tension glaucoma and primary open-angle glaucoma (P < 0.001 for XFG, P < 0.05, and P < 0.01 for SOAG, respectively). ^*^P < 0.05, ^**^P < 0.01, and^***^P < 0.001. The correlation between the total sum of needlings and the aqueous ATX levels at 1 **(C)**, 3 **(D)**, 6 **(E)** and 12 **(F)** months after trabeculectomy. At 3 **(D)**, 6 **(E)** and 12 **(F)** months after surgery, the number of needlings showed a statistically significant correlation between the levels of ATX in the AH, but there was no significant correlation between the total sum of needlings and the AH ATX levels at 1 month **(C)**.

A total of 70 glaucomatous eyes of 70 patients, including 14 normal-tension glaucoma (NTG), 29 POAG, 15 SOAG, and 12 exfoliative glaucoma (XFG) eyes were included in the study. Demographic characteristics of the study population are listed in Table 1. The preoperative IOP showed higher values for SOAG compared to other groups (P < 0.0001; Table 1). There was a significant correlation between the NTG and POAG, NTG and SOAG, and POAG and SOAG groups with a history of phacoemulsification (P < 0.0001). Comparison between different patient groups showed that the ATX level was significantly higher in the XFG group compared to other groups (P < 0.05; Fig. 1A). Total LPA was also significantly higher in the XFG and SOAG groups compared to the NTG and POAG groups (P < 0.001 for XFG, P < 0.05 and P < 0.01 for SOAG, respectively), whereas the differences between SOAG and XFG groups showed no statistical significance (Fig. 1B).

**Table 1:** Demographic characteristics of the study population.

Variables	NTG	POAG	SOAG	XFG	P-value^‡^
Patients (No.)	14	29	15	12	
Sex (male: female)	7:7	19:10	8:7	8:4	NS*
Age, mean (SD), years	64.8 (10.3)	66.0 (12.0)	62.3 (13.4)	74.3 (8.7)	NS** (P = 0.0566)
[range]	49–80	42–84	37–82	61–86	
Preoperative IOP, mean (SD), mmHg	15.9 (2.9)	20.7 (4.9)	31.2 (11.7)	23.8 (8.7)	^†^<0.0001***
[range]	9–21	14–33	12–49	13–43	
MD, mean (SD), dB	−18.2 ± 6.6	−19.37 ± 6.3	−16.6 ± 8.4	−15.8 ± 7.7	NS**
[interquartile]	−29.5 to −4.3	−30.5 to −9.9	−28.5 to −1.85	−26.9 to −2.7	
Glaucoma eye drops, mean (SD), No.	3.1 (0.9)	3.8 (1.0)	3.7 (0.8)	4.0 (0.7)	NS**
[range]	2–5	1–5	2–5	3–5	
History of phacoemulsification, No. (%)	3 (21)	7 (24)	7 (47)	6 (50)	<0.0001*

NTG, normal-tension glaucoma; POAG, primary open-angle glaucoma; SOAG, secondary glaucoma; XFG, exfoliative glaucoma; IOP, intraocular pressure.

^*^Fisher’s exact test; ^**^analysis of variance; ^***^Kruskal-Wallis; ^†^statistically significant difference from the SOAG group (Steel-Dwass test); ^‡^comparison of the four glaucoma subtypes.

### AS-OCT of bleb formation

**Figure 2:**
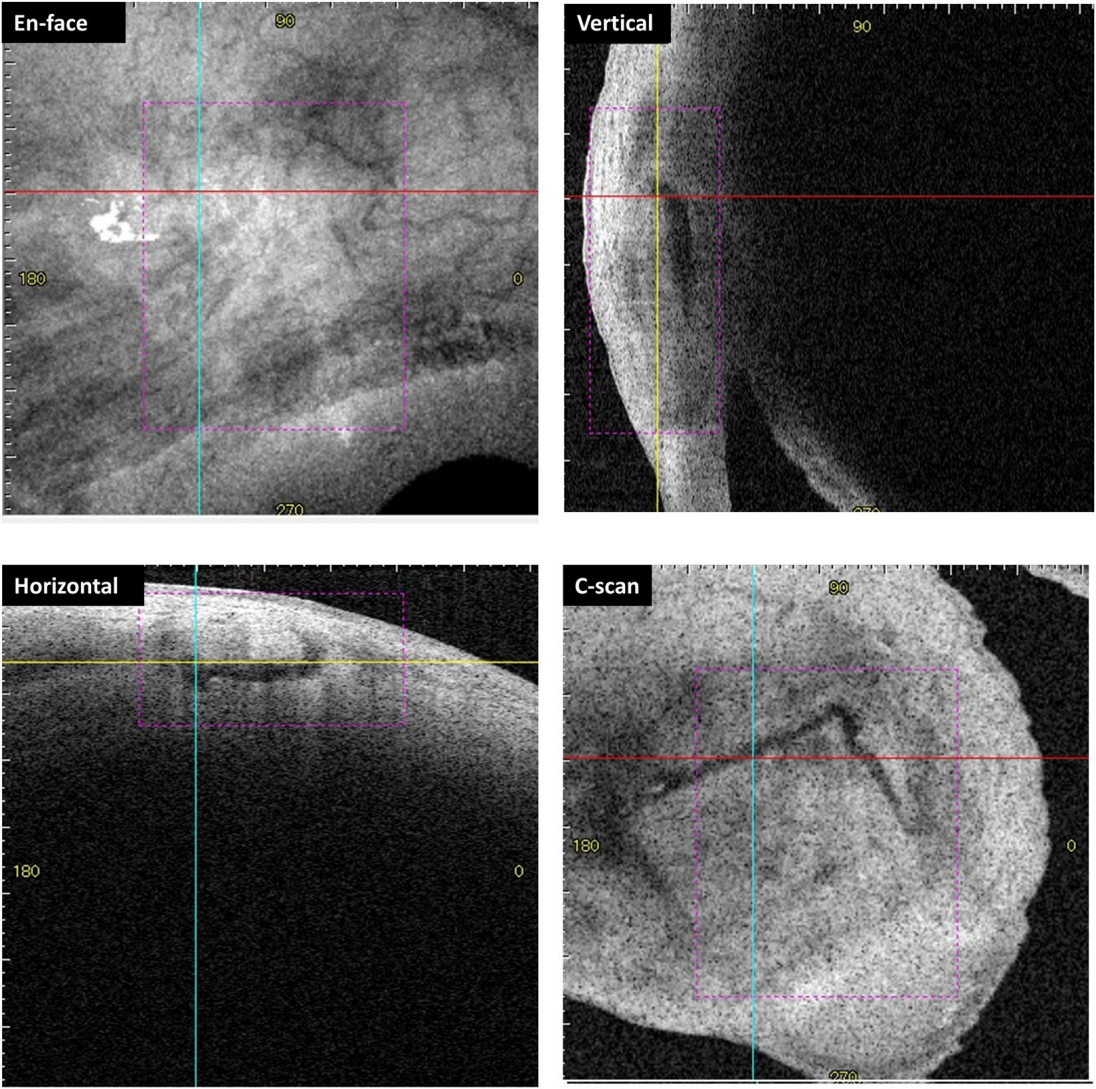
Identifying the filtration openings on scleral flaps using three-dimensional anterior segment optical coherence tomography. An example of a measurement of the bleb wall thickness (82-year-old, primary open-angle glaucoma, 6 months post-surgery). En face photograph is shown in the top left, the vertical image is shown in the top right, the horizontal image is shown in the bottom left, and the C-scan image is shown in the bottom right. The red and blue lines indicate horizontal and vertical axes, respectively. The yellow lines indicate the Z-axis of the data corresponding to the C-scan images.

The center of the filtration opening points were identified using vertical and horizontal scans, and checked using the C-scan image, which visualized the scleral flap (Fig. 2). There were 26 eyes whose filtration opening points were detectable at 1 month after surgery, 34 eyes at 3 months, and 22 eyes at 6 months. Scleral flap adhesion or bleb localization were identified in four eyes at 1 month, 12 eyes at 3 months, and 19 eyes at 6 months after surgery. Median bleb depth was 0.33 mm, 0.28 mm and 0.26 mm for 1,3, and 6 months after operation (Table 2). Median bleb density was 142.3, 141.3 and 137.3 for 1,3, and 6 months after operation (Table 2). Vascularity scoring referring to Moorfields Bleb Grading showed significant relevance to postoperative IOP at 1, 3 and 12 months after operation (Fig, 3A,B,D). There were no significant correlations between bleb depth/bleb density/vascularity scoring and glaucoma subtypes or ATX/LPA levels (see Supplemental Tables S1 and S2).

**Table 2:** Assessment of bleb morphology according to AS-OCT examination and vascularity scoring.

	At 1 month	At 3 months	At 6 months	At 12 months	p value^‡^
Bleb depth, mean (SD), mm	0.33 (0.24)	0.28 (0.27)	0.26 (0.34)	—	NS
[range]	0.24–0.42	0.2–0.36	0.15–0.37	—	NS
Bleb density, mean (SD), optical density unit	142.3(32.6)	141.3(32.6)	137.3(33.8)	—	NS
[range]	130.1–154.5	131.7–150.9	126.7–148.0	—	NS
Vascularization, mean (SD), grading	2.7 (1.2)	2.4 (1.2)	2.2 (1.1)	2.0 (0.7)	NS
[range]	1~5	1~4	1~4	1~4	NS

^‡^Comparison of the four glaucoma subtypes (NTG, POAG, SOAG, XFG).

**Figure 3:**
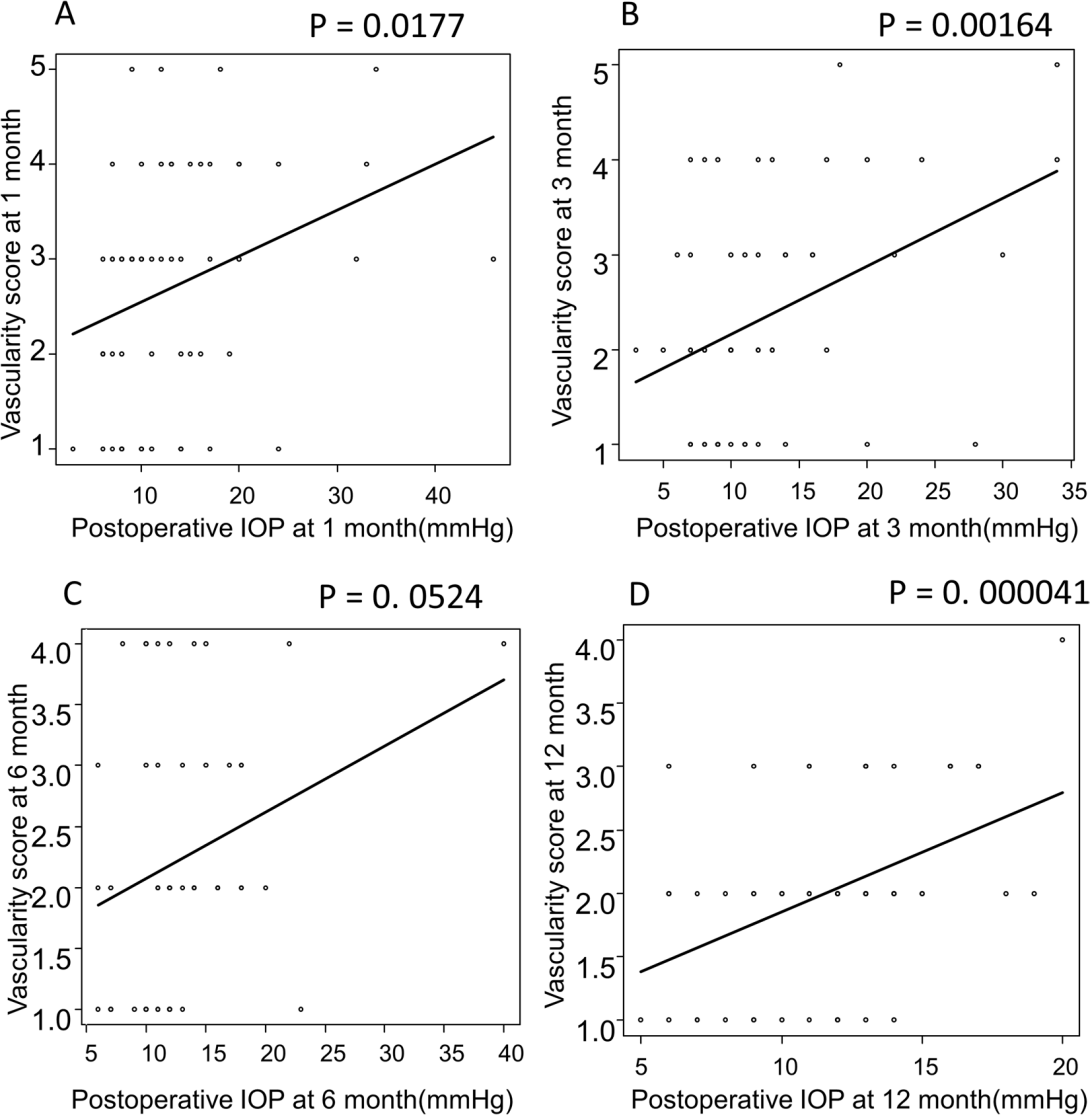
Relationships between postoperative IOP and the vascularity score of the bleb according to Moorfields Bleb Grading. Relationships between postoperative IOP and vascularity scoring at 1 **(A)**, 3 **(B)**, 6 **(C)** and 12 **(D)** months post-surgery. Postoperative IOP and bleb vascularity showed significant correlation at 1 **(A)**, 3 **(B)** and 12 **(D)** months. There was no significant correlation at 6 **(C)** months after surgery.

### Correlation between needling and bleb formation and postoperative IOP

**Figure 4:**
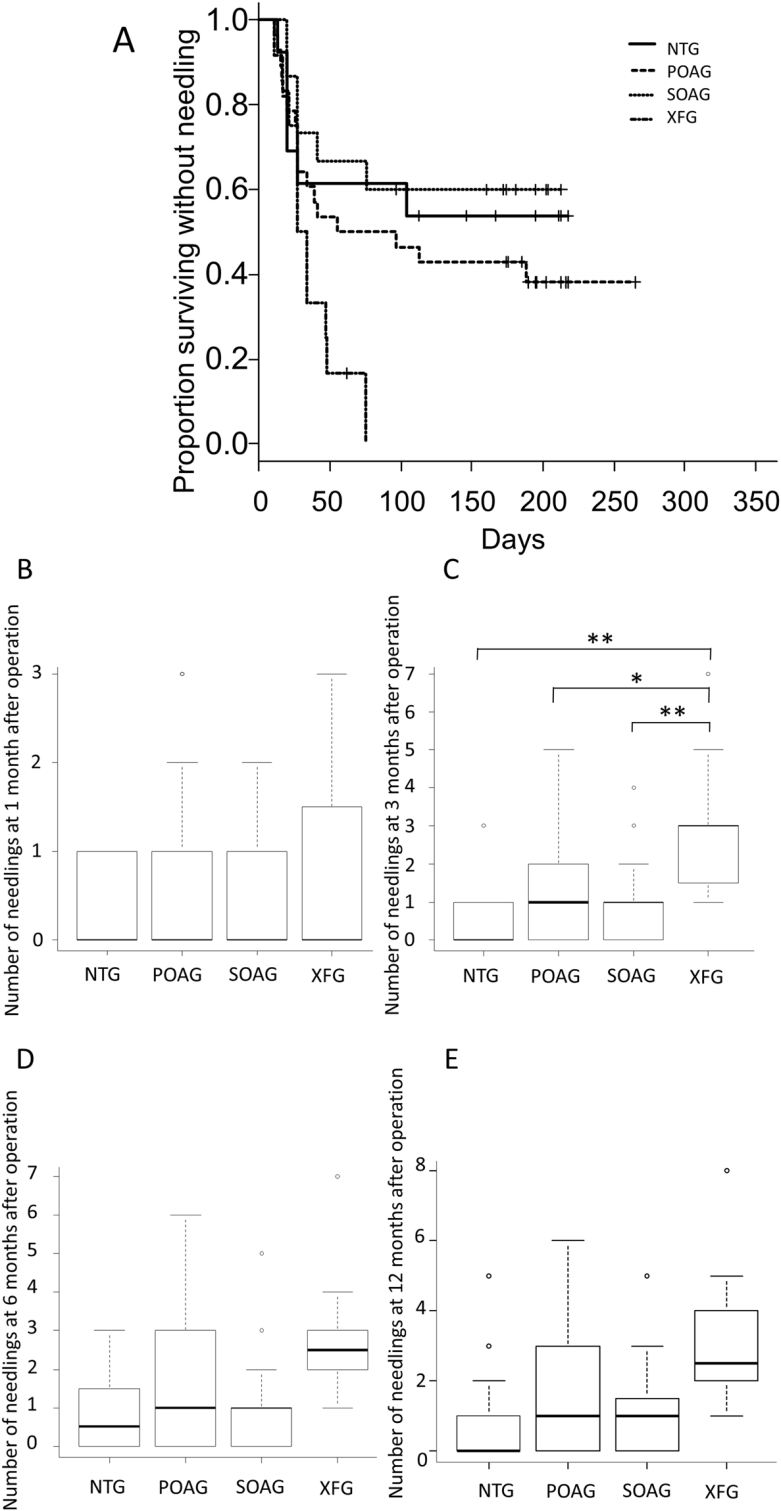
Time course of cumulative success rate without needling and the total sum of needlings after operation according to the glaucoma subtypes. **(A)** The cumulative success rate of trabeculectomy without needling according to the glaucoma subtypes was determined by Kaplan–Meier analyses. The cumulative complete success rate was significantly lower in the XFG group than in any other glaucoma group (log-rank test; P = 0.0262). The total sum of needlings at 1 **(B)**, 3 **(C)**, 6 **(D)** and 12 **(E)** months after trabeculectomy according to glaucoma subtypes. There was no significance among the glaucoma groups for the total sum of needlings at 1 **(B)**, 6 **(D)** and 12 **(E)** months after surgery. **(C)** The total sum of needlings was significantly higher in the exfoliative glaucoma group compared to all of the other glaucoma groups at 3 months after surgery (P < 0.01). ^*^P < 0.05 and ^**^P < 0.01.

Postoperatively, needling was performed following the criteria mentioned in the Patients and Methods section, and the cumulative number of eyes treated with the needling was 25 eyes at 1 month, 41 eyes at 3 months, and 43 eyes at 6 and 12 months after surgery. The total sum of needlings for each glaucoma subtypes at 1, 3 and 6 and 12 months after surgery were shown in Fig. 4B–E. The total sum of needlings was significantly higher in the XFG group compared to all of the other glaucoma groups at 3 months after surgery (Fig. 4C; P < 0.01). Correlation between the total sum of needlings and aqueous ATX levels at 1, 3, 6 and 12 months after operation are shown in Fig. 1C–F. At 3, 6 and 12 months after surgery, the number of needlings showed a statistically significant correlation between the levels of ATX in the AH (Fig. 1D–F). Multivariate analyses for factors associated with the presence of needling showed that total ATX, which was significantly higher in the XFG group preoperatively, showed significance at 1, 3, 6 and 12 months (Table 3).

**Table 3:** Multivariate analyses for factors associated with the presence of needling at 1, 3 and 6 months after surgery.

Parameters	At 1 month		**At 3 months**		At 6 months		At 12 months	
	β	p value	β	p value	β	p value	β	p value
Age.y	0.013	**0**.0346*	—	—	—	—	—	—
IOL (phakia vs.pseudophakia)	—	—	—	—	—	—	—	—
pre IOP	—	—	—	—	—	—	—	—
Glaucoma subtypes (NTG, POAG, SOAG, XFG)	—	—	—	—	—	—	—	—
Total ATX	0.404	**0**.0446*	0.587	**0**.003**	0.493	**0**.0096**	0.526	**0**.008*
Total LPA	—	—	—	—	—	—	—	—

IOL, intraocular lens; PE, pseudo exfoliation; NTG, normal-tension glaucoma; POAG, primary open-angle glaucoma; SOAG, secondary glaucoma; XFG, exfoliative glaucoma; IOP, intraocular pressure; ATX, autotaxin; LPA, lysophosphatidic acid.

### The success percentage after trabeculectomy

Next, we evaluated the success percentages after trabeculectomy with follow up period up to 12 months, without needling, according to different glaucoma subtypes, because postoperative needling was frequently performed in the XFG group. The cumulative complete success percentage was significantly lower in the XFG group than in any other glaucoma group (log-rank test; P = 0.0262; Fig. 4A).

### The effects of ATX and ATX inhibitor on mRNA expression of fibronectin and COL1A1 in HCFs

**Figure 5:**
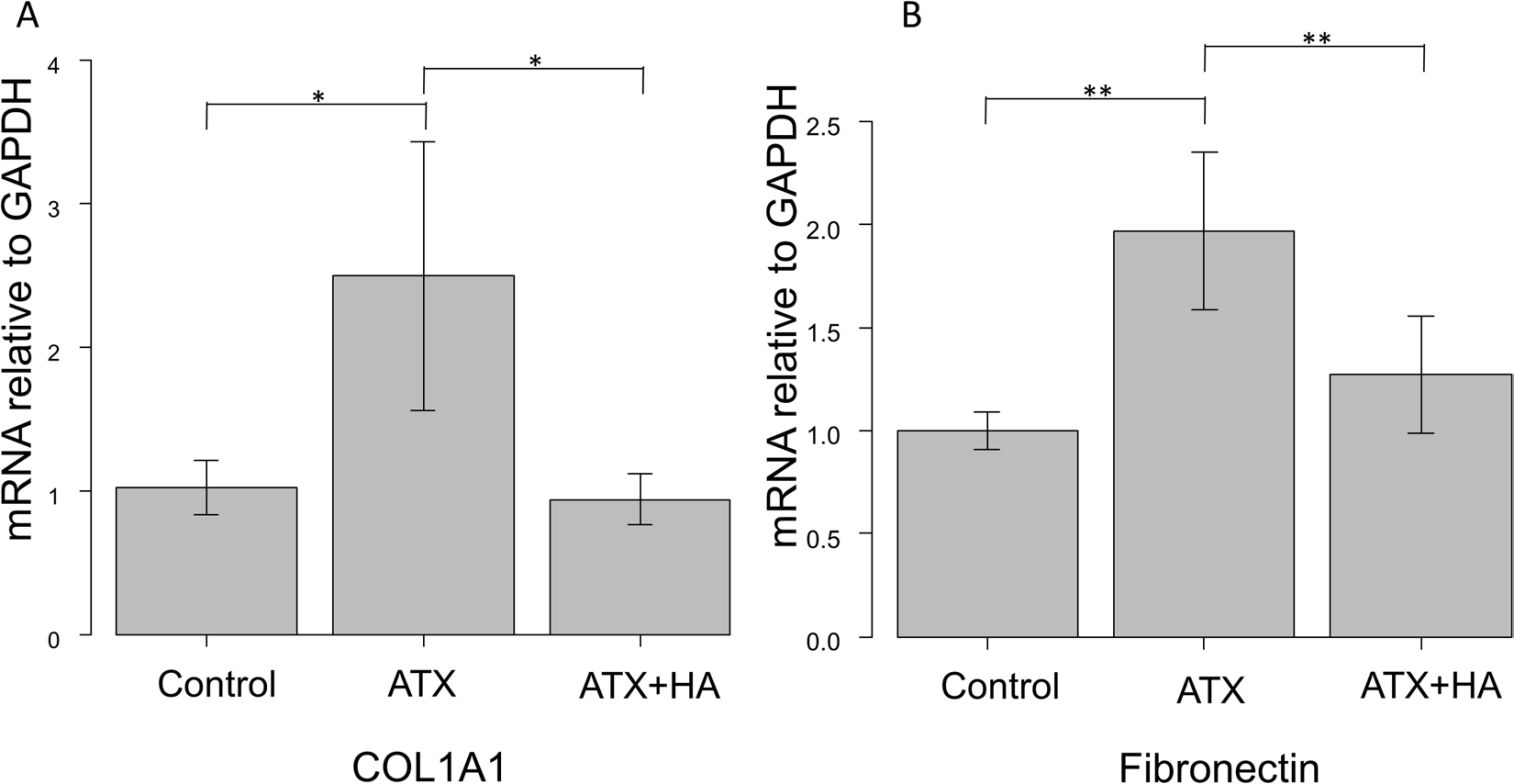
qPCR quantification of the human conjunctiva fibroblast mRNA expression of COL1A1 and fibronectin relative to GAPDH (n = 4). The relative mRNA expression of **(A)** COL1A1 and **(B)** fibronectin was significantly higher compared to the control groups when stimulated with ATX, and the expression was inhibited by treatment with its inhibitor. ^*^P < 0.05 and ^**^P < 0.01.

The mRNA levels in HCFs were assessed by qPCR (Fig. 5A,B). The relative mRNA expression of fibronectin and COL1A1 was significantly higher compared to the control groups when stimulated with ATX, and the expression was inhibited by the ATX inhibitor.

### The effects of ATX and ATX inhibitor on ATX-induced fibrotic responses in HCFs assessed by immunohistochemistry and western blotting

**Figure 6:**
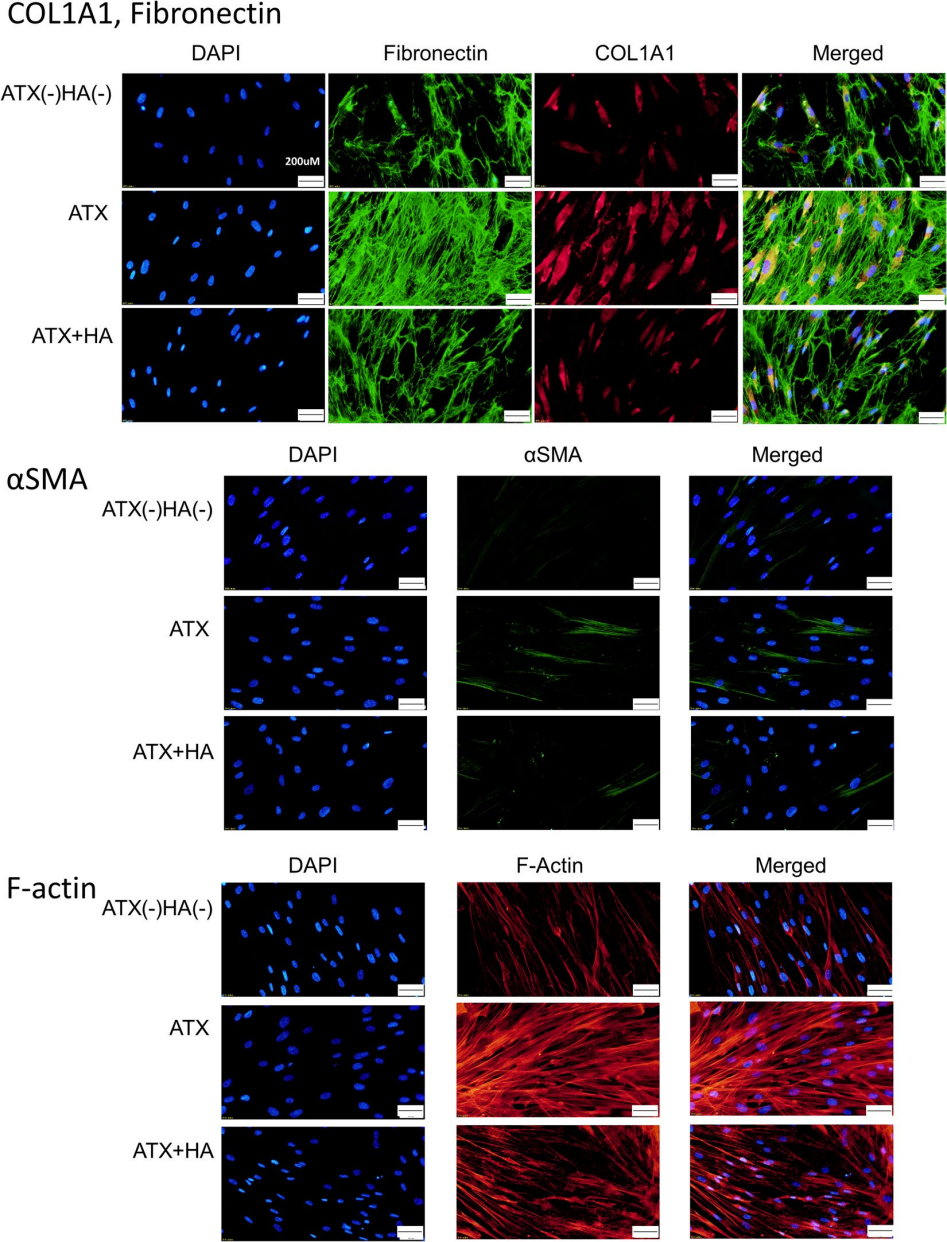
Immunocytochemistry of fibronectin, COL1A1, F-actin and αSMA in HCFs. The left panels show cells that were stained with DAPI. The middle panels show cells stained for either COL1A1, fibronectin, F-actin orαSMA. The right panels show merged image. The expression of COL1A1 and fibronectin is shown in the upper row, αSMA in the middle row, and phalloidin in the lower row. These components were upregulated in HCFs with ATX treatment, and the fibrotic responses were significantly suppressed by HA130 treatment. Bar, 200 μm.

**Figure 7:**
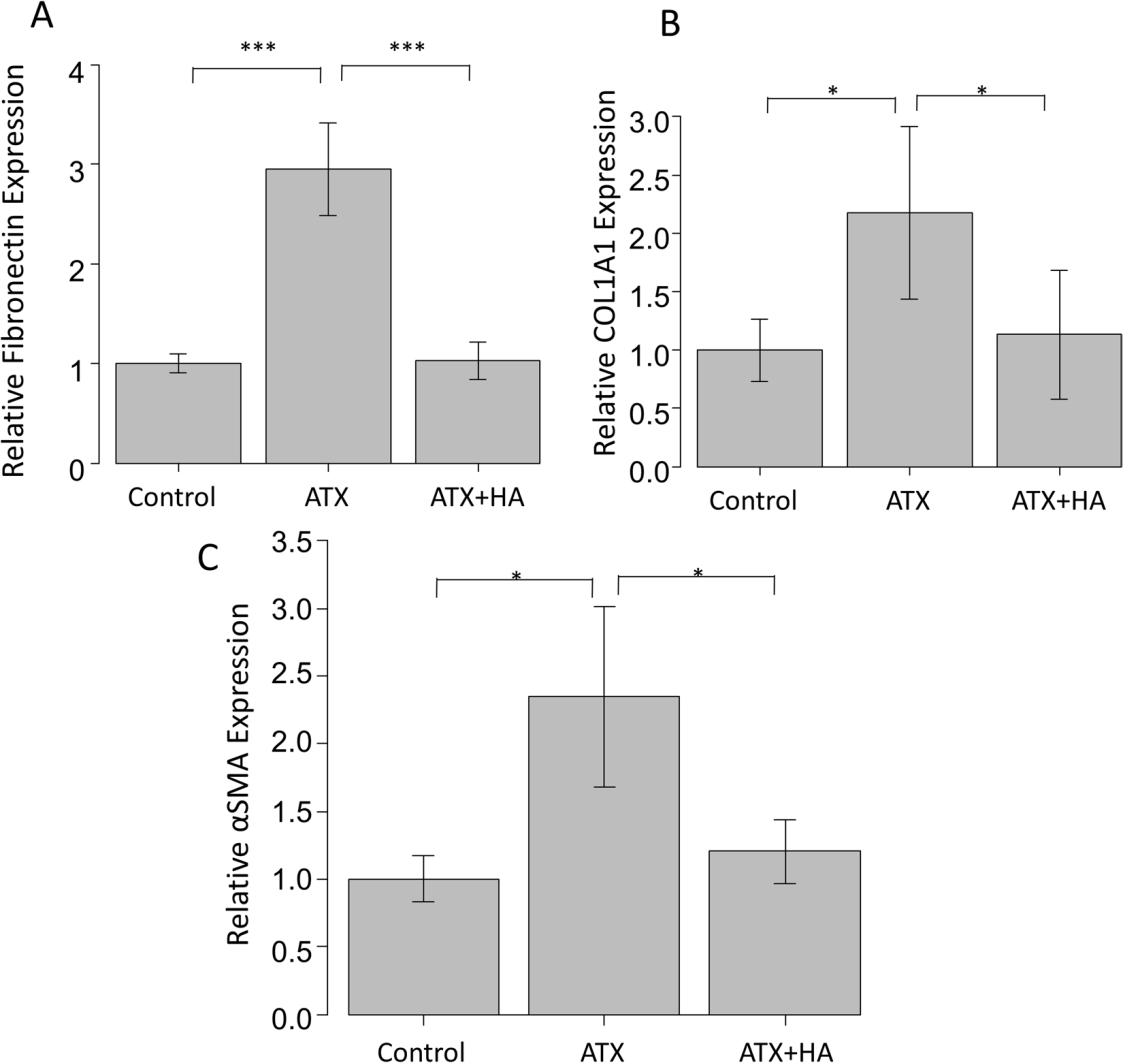
Western blotting of fibronectin, COL1A1, F-actin and αSMA in HCFs. **(A**–**C)** shows the relative expression of fibronectin, COL1A1 and αSMA (n = 3). Results are expressed relative to the loading control (β-tubulin). These components were upregulated in HCFs with ATX treatment, and the fibrotic responses were significantly suppressed by HA130 treatment. ^*^P < 0.05, ^**^P < 0.01 and ^***^P < 0.001.

Immunocytochemistry and western blotting were used to assess the fibrotic responses to ATX and the effects of the ATX inhibitor, HA130. Figure 6 shows the immunocytochemistry that the expression of fibronectin, COL1A1, phalloidin and αSMA was upregulated in HCFs after ATX treatment, and these fibrotic responses were significantly suppressed by HA130 treatment. Figure 7 shows the data from western blotting (n = 3), also showing that the expression of fibronectin, COL1A1 and αSMA was upregulated in HCFs after ATX treatment, and these fibrotic responses were significantly suppressed by HA130 treatment.

## Discussion

It is important to prevent scarring after trabeculectomy, because fibrosis of blebs often leads to surgical failure, which has been reported in 35–43% of cases due to postoperative scarring and subconjunctival fibrosis^24,25^. Many procedures including adjunctive treatment with antiproliferative agents such as MMC, 5-fluorouracil, or anti-TGF-β antibodies have tried to prevent scarring and bleb failure, but these treatments have failed to maintain tissue health, and often led to thin-walled blebs with a risk for bleb leakage and infection, without facilitating the successful formation of glaucoma filtration blebs^24–26^.

Various mediators including TGF-β, VEGF, CTGF, MCP-1 and members of the MMP family are involved in the pathogenesis of scarring after trabeculectomy^11–16,27^. Among these growth factors, the levels of several factors such as TGF-β, VEGF, CTGF, and MCP-1 increased in the AH in POAG, XFG, or neovascular glaucoma patients, which suggests that these factors stimulated the signaling pathway leading to myofibroblast transdifferentiation of HCFs, monocyte-derived cell infiltration, and ECM remodeling after trabeculectomy^11,13–15,27^. Several *in vivo* and *in vitro* studies have suggested that modulation of these factors could be used as a novel strategy to control scarring after trabeculectomy^13,14,28^.

In this study, we investigated the correlation between ATX and LPA levels in the AH and the formation of functional filtration blebs using AS-OCT after trabeculectomy. Our results showed a significant increase in ATX and LPA levels in the SOAG and XFG groups, which significantly correlated with glaucoma subtype in good accordance to our previous report^23^ (Fig. 1A,B). The aqueous ATX levels significantly correlated with the presence of needling, and the total sum of needlings (Fig. 1D–F; Table 3). Figure 4C shows that among glaucoma subtypes, the XFG group, whose ATX levels were significantly high, showed a significantly increased number of needling at 3 months after surgery, and the cumulative success rate without needling was significantly lower in this group compared to other glaucoma subtypes (Fig. 4A).

Though neither bleb depth nor bleb density showed relativity to post-operative IOP, vascularity scoring referring to Moorfields Bleb Grading showed significant relevance to postoperative IOP at 1, 3 and 12 months after operation (Fig. 3A,B,D).

After tissue damage, activated fibroblasts migrate to the damage site, where they differentiate into αSMA-positive myofibroblasts, which synthesize ECM components such as collagen, and contribute to the formation of contractile stress fibers that bind to the ECM^29^. LPA has been recognized as a major bioactive lipid mediator that influences fibrosis, and induces many kinds of cellular responses including Rho GTPase-regulated cell adhesion, contraction, cellular proliferation, prevention of apoptosis, cell migration, cytokine and chemokine secretion, platelet aggregation, transformation of smooth muscle cells, and neurite retraction^30,31^. We previously reported that LPA stimulated HCFs, resulting in αSMA overexpression, fibrosis, and ECM deposition^17^. In this study, aqueous LPA levels were significantly higher in SOAG and XFG groups compared to the NTG and POAG groups. Therefore, aqueous LPA could be a potent factor that stimulates fibrosis by inducing HCF migration, proliferation, and ECM deposition, particularly in SOAG and XFG eyes.

ATX, the enzyme generating enzyme LPA, is involved in fibrotic changes in many organs and diseases^32–34^. We have recently reported that the aqueous ATX was involved in the fibrotic changes of trabecular meshwork cells, and ATX was highly expressed in the trabecular meshwork of trabeculectomy specimens^23^. In the present study, we compared the levels of ATX in the AH according to the glaucoma subtypes and found that the XFG group showed significantly higher ATX concentrations (Fig. 1A), and multivariate analyses showed that ATX levels were a significant factor for the presence of needling at 1, 3, 6 and 12 months after surgery (Table 3). We also showed a significant correlation between the concentration of ATX and the total sum of needling within 12 months after surgery (Fig. 1D,E,F). Furthermore, the cumulative success rate without needling was significantly lower in the XFG group whose ATX levels were significantly higher (Fig. 4A). These findings suggested that the preoperative levels of ATX in the AH, and the resultant LPA formation could be important factors involving formation of filtration blebs, which could cause excessive fibrotic changes in XFG eyes.

Previous studies have reported that filtration surgery failure was particularly high in the XFG group^35,36^, because XFG causes more severe inflammation and excessive fibrosis compared to other glaucoma subtypes^36–38^. Similar to the previous studies, in our present study, the increased fibrotic changes occurred especially in XFG patients, with higher aqueous ATX levels and a low success rate, which required more number of postoperative needling. Figure 4C suggests that the total sum of needlings in the XFG group was very different than that for the other glaucoma subtypes, because fibrotic changes at filtration points occurred more often because of excessive inflammation in XFG patients. Based on these results, it was speculated that the more the necessary needling needed and the more the severe inflammation occurred, conjunctival health, bleb function and vascularization of the bleb wall were impaired and resulted in a non-functional bleb. Proinflammatory mediators such as TGFβ, IL-6, and IL-8 are reported to be involved in the pathogenesis of XFG^36–40^, and our present result suggested that ATX may also be an important factor, which stimulates the inflammatory and fibrotic phase after glaucoma filtration surgery. ATX inhibitors have recently been developed and suggested as possible treatments for excessive inflammation^41^. We speculated that the ATX inhibitor could be useful for the inhibition of the excessive fibrogenic changes and inflammation, and improvement of the surgical outcome of filtering surgery.

Therefore, in addition to these clinical results, we conducted *in vitro* studies to determine the effects of ATX in fibrotic changes in HCFs (Figs 5A,B, 6 and 7A–C). ATX treatment significantly induced fibronectin and COL1A1 expressions in HCFs, which was shown by qPCR and immunocytochemistry and western blotting (Figs 5A,B, 6 and 7A–C). The ATX inhibitor, HA130, significantly suppressed these fibrotic responses (Figs 5A,B, 6 and 7A–C), which suggested that the inhibition of ATX could be a target for inhibiting fibrosis after trabeculectomy.

This study had several limitations. Blood contains high amounts of ATX, so the relatively high ATX levels in SOAG, XFG, and pseudophakic eyes may have been caused by a breakdown of the blood-aqueous barrier. Further studies are needed to determine the origin of the aqueous ATX levels. We were also unable to evaluate the AH after surgery; therefore, it was unknown whether preoperative ATX levels persisted for extended times after surgery. We need to assess the ATX levels in the AH postoperatively to determine whether the high ATX levels are sustained and influence fibrosis after surgery in the future study. Moreover, we could not characterize the downstream signaling cascade of the ATX-LPA pathway in detail. However, Rho-ROCK signal pathway is one of the downstream cascade of the ATX-LPA pathway which we also have previously reported to be involved in the fibrosis after glaucoma surgery^17^, and which we believe it to be a supportive data for the present results. Regarding the effects of ATX and ATX inhibition on fibrotic changes in HCFs, the results of ECM changes using culture cells in the present study are not sufficient enough to conclude an anti-fibrotic effect of ATX inhibitor, and an *in vivo* study using an animal model of filtering surgery or clinical studies should be performed to assess the clinical relevance of ATX and its inhibition in trabeculectomy.

In glaucoma surgery, postoperative fibrosis or scarring at the surgical site and subconjunctival spaces continues to be a major obstacle to achieve sustained and effective IOP lowering. In the present study, we found that the ATX-LPA pathway was a target for downregulating the fibrogenic cascade. Modulation of ATX-LPA pathway could be used as a novel modality to inhibit the excessive fibrogenic changes in filtration blebs and improve surgical outcome after glaucoma surgery.

## Patients and Methods

### Patients and samples from patients who underwent glaucoma surgery

AH samples were obtained from glaucoma patients who underwent trabeculectomy from January to May in 2016 at the University of Tokyo Hospital. This prospective observational study was approved by the Institutional Review Board of the University of Tokyo and was registered with the University Hospital Medical Information Network Clinical Trials Registry of Japan (ID: UMIN000027137). All of the procedures conformed to the Declaration of Helsinki. Written informed consent was obtained from each patient. The patients’ characteristics are shown in Table 1. Glaucoma patients ≥20 years old, who underwent trabeculectomy for open-angle glaucoma (OAG), were recruited. Exclusion criteria included patients with other types of glaucoma including primary angle-closure glaucoma, congenital/developmental glaucoma, and patients with a previous history of intraocular surgery other than small incision cataract surgery without complications. POAG patients had a glaucomatous visual field or optic disc, as well as a normal angle with gonioscopy. If untreated IOP was below 21 mmHg, the patients were defined as having NTG. OAG patients with inflammatory diseases or uveitis were recruited as SOAG and those with exfoliative material were classified as having XFG. The IOP was determined using a Goldmann tonometer, and the maximum preoperative IOP was evaluated within 3 months prior to surgery. When both eyes of a patient met the inclusion criteria, only the eye treated first was included in the analyses. For all of the patients, the anterior eye segment and optic disc were examined by glaucoma specialists using a slit-lamp biomicroscope and dilated fundoscopy to diagnose glaucoma.

### Surgical procedures and postoperative management

Three experienced surgeons performed all of the trabeculectomies as follows. The preoperative AH was obtained at the start of the surgery before any incisional procedures, using limbal paracentesis and a syringe and 30-gauge needle. Approximately 70–100 μL was collected in a PROTEOSAVE SS 1.5 mL Slimtube (Sumitomo Bakelite, Tokyo, Japan), registered, and stored at −80 °C until processing. The bleb was formed using a fornix-based procedure, and a square half-layer scleral flap (3 mm width) was created and exposed to 0.4 mg/mL mitomycin C for 3 min, followed by washing with 100 mL balanced saline solution. Then a corneoscleral block was excised to create a fistula, which was followed by peripheral iridectomy. The tenons layer was mobilized during the procedure. Then the scleral flap and conjunctiva were sutured with 10–0 Nylon to prevent leakage.

We aimed to control post-operative intraocular pressure (IOP) less than 12 mmHg, and needling was performed when the IOP elevated with scleral flap adhesion or the localization of the bleb. Also, if the IOP elevated larger than pre-operative state in cases with preoperative IOP levels lower than 12 mmHg, needling procedure was added. All of the patients were treated postoperatively with 0.1% topical betamethasone and 0.5% moxifloxacin for approximately 3 months. Postoperative IOPs at 1, 3, and 6 months were recorded at the outpatient clinic before the AS-OCT, because the eyelid had to be lifted using a finger before AS-OCT.

### AS-OCT measurements

Bleb morphology and parameters were evaluated using photographs and three-dimensional AS-OCT (CASIA; Tomey, Nagoya, Japan) at 1, 3, and 6 months after surgery as previously described^42^ to assess the bleb formation and determine whether the bleb needs needling. AS-OCT was captured after careful elevation of the upper eyelid with the examiner’s finger, to expose the bleb. All of the data were assessed with either vertical or horizontal sections to identify the opening point of the scleral flap (Fig. 2). If the fluid cavity of the bleb was filled and we did not find the opening point with vertical or horizontal scans, a C-scan was used to find the opening points. If the bleb was not filled with fluid and adhered to the sclera, it was regarded as scleral adhesion occurring or the bleb is starting to be localized. Results of the aqueous ATX or LPA levels were masked from the clinicians who examine AS-OCT. Then correlations of these findings with aqueous ATX levels and other clinical data were determined. Also, bleb depth and density were assessed referring to the previous report^43^. And the vascularity of the bleb were scored according to the Moorfields Bleb Grading (Grading 1–5)^9^. Those bleb morphology and relativity to IOP, glaucoma subtypes and ATX/LPA levels were also examined.

### Measurements of LPA using LC-MS/MS

Quantification of the lysophospholipids was performed as previously described^24,44^. Briefly, ten μL AH sample per patient was used for identification and quantification of lysophospholipids. AH samples were mixed with a 10-fold volume of methanol and an internal standard and then sonicated. After centrifugation at 21,500 × *g*, the resulting supernatant was recovered and used for LC-MS analyses. Twelve acyl chains (14:0, 16:0, 16:1, 18:0, 18:1, 18:2, 18:3, 20:3, 20:4, 20:5, 22:5, and 22:6) were monitored and the total LPA was calculated.

### Measurement of ATX and ATX isoforms

The ATX levels in the AH were determined using a two-site immunoenzymatic assay with an ATX assay reagent equipped with a Tosoh AIA system (Tosoh, Tokyo, Japan) as previously described^23,45–48^.

### Cell culture and passage

HCF cells were purchased from ScienCell Research Laboratories (San Diego, CA, USA) and cultured in Fibroblast Medium (ScienCell Research Laboratories) with Fibroblast Growth Supplement to ascertain the cell phenotypes during passages. Cells from passages 3–6 were used for the experiments. The cells were treated with 44 μM ATX for 6 h with or without pretreatment for 30 min with 1 µM HA130, an ATX inhibitor (Santa Cruz Biotechnology, Santa Cruz, CA, USA).

### Quantitative PCR

The cells were lysed using ISOGEN (NIPPON GENE, Tokyo, Japan), and mRNA was isolated using chloroform and isopropyl alcohol. The mRNA was treated with a PrimeScript RT Reagent Kit (Takara Bio, Shiga, Japan) to synthesize the cDNAs. The mRNA levels were quantified using quantitative PCR (qPCR) of cDNA with SYBR Premix Ex Taq II (Tli RNaseH Plus) (Takara) and the Thermal Cycler Dice Real Time System II (Takara) with the ΔΔCt method. For qPCR, primer sequences for GAPDH and fibronectin were taken from previously published sequences, and the primers were purchased from Hokkaido System Science (Hokkaido, Japan).

The sequences of the PCR primers were GAPDH: forward, 5′-GAGTCAACGGATTTGGTCGT-3′ and reverse, 5′-TTGATTTTGGAGGGATCTCG-3′; fibronectin: forward, 5′-AAACCAATTCTTGGAGCAGG-3′ and reverse, 5′-CCATAAAGGGCAACCAAGAG-3′; and collagen type I, alpha 1 chain (COL1A1): forward, 5′-CAGCCGCTTCACCTACAGC-3′ and reverse, 5′-TTTTGTATTCAATCACTGTCTTGCC-3′. The data were normalized relative to GAPDH.

### Immunocytochemistry

Cells were grown in chamber slides. After serum starvation for 24 h, the cells were treated with 44 µM ATX for 24 h, with or without the ATX inhibitor, HA 130 (1 µM), which was added 30 min before ATX treatment. The cells were fixed in ice-cold 4% paraformaldehyde for 15 min, permeabilized with 0.3% Triton X-100 for 5 min, and blocked in 3% bovine serum albumin for 30 min. The primary antibodies were anti-fibronectin (1:400; Abcam, Cambridge, MA, USA), anti-collagen type I (1:400; Cell Signaling Technology, Danvers, MA, USA), anti-rhodamine phalloidin (7:1000; Thermo Fisher Scientific, Waltham, MA, USA), and anti-αSMA (Sigma-Aldrich Co., LLC St. Louis, MO USA, 1:500). Alexa Fluor 488 and 594 secondary antibodies (1:1,000) were purchased from Thermo Fisher Scientific.

### Western blotting

Cells were first starved by incubation for 24 hours in serum-free medium. After serum starvation for 24 h, the cells were treated with 44 µM ATX for 24 h, with or without the ATX inhibitor, HA 130 (1 µM), which was added 30 min before ATX treatment. After 24 hrs of incubation, cells were collected in RIPA Buffer (Thermo Fisher Scientific K.K., Kanagawa, Japan) containing protease inhibitors (Roche Diagnostics, Basel, Switzerland), sonicated, and centrifuged. Protein concentrations in the supernatant were determined by a BCA assay using a BCA Protein Assay Kit (Thermo Fisher Scientific K.K., Kanagawa, Japan). Proteins were boiled in Sample Buffer (Thermo Fisher Scientific K.K., Kanagawa, Japan). The same amounts of proteins were loaded onto 4–20% precast polyacrylamide gels (BIO-RAD Laboratories, Hercules, CA USA) and separated by SDS-PAGE. Protein bands were transferred to PVDF membranes (BIO-RAD Laboratories, Hercules, CA USA) and the membranes were immersed in Tris-Buffered Saline with Tween 20 (TBST) containing the first antibody. After washing, the membranes were immersed in TBST containing the second antibody and reacted with ECL substrate (Thermo Fisher Scientific K.K., Kanagawa, Japan). Protein bands were detected by ImageQuant LAS 4000 mini (GE Healthcare, Chicago, IL USA). Primary antibodies were: anti-αSMA (Sigma-Aldrich Co., LLC St. Louis, MO USA, 1:1000) and anti-fibronectin (1:1000; Abcam, Cambridge, MA, USA), anti-collagen type I (1:1000; Cell Signaling Technology, Danvers, MA, USA). HRP-conjugated second antibody (1:10,000) was purchased from Thermo Fisher Scientific (Waltham, MA USA). β-tubulin served as the loading control. All membranes were stripped of antibodies using WB Stripping solution and incubated with mouse monoclonal antibody β-tubulin (1:1000), and subsequently with H goat anti-mouse IgG antibody (1:2000). Densitometry of scanned films was performed using ImageJ 1.49 (NIH Bethesda, MD, USA), and results are expressed relative to the loading control (β-tubulin).

### Statistical analysis

Data were statistically analyzed using the EZR program (Saitama Medical Center, Hidaka, Japan)^49^. The results were expressed as the mean ± standard deviation (SD). The Mann-Whitney U test and chi-square or Fisher’s exact test were used for comparing two variables, and the Steel-Dwass test was used for multiple variables. According to the Kolmogorov-Smirnov and Levene tests, nonparametric tests were used, because most clinical variables failed to satisfy normality or equality of variance assumptions. Spearman’s correlation test was used to assess the correlations. The independent effects of the LPC levels, ATX levels, and other background characteristics of IOP or glaucoma diagnoses were evaluated using simple and stepwise multiple linear regression analyses. Differences in the data among the groups were analyzed by one-way analysis of variance and Tukey’s test as a *post hoc* test. A value of P < 0.05 was considered statistically significant.

## Electronic supplementary material

Supplemental Table S1 and S2
