# Supplementary material for: Increased aqueous autotaxin and lysophosphatidic acid levels are potential prognostic factors after trabeculectomy in different types of glaucoma
Source: Sci Rep. 2018 Jul 27;8:11304. doi: 10.1038/s41598-018-29649-3 (PMC6063955; doi:10.1038/s41598-018-29649-3)
Supplement: Supplementary file 1 — Supplemental Table S1 and S2 [file 41598_2018_29649_MOESM1_ESM.docx]

**Increased aqueous autotaxin and lysophosphatidic acid levels are potential prognostic factors after trabeculectomy in different types of glaucoma**

Nozomi Igarashi^1^ M.D, Megumi Honjo^1^ M.D, Ph.D., Makoto Kurano^2,3^ M.D, Ph.D., Yutaka Yatomi^2,3,4^ M.D, Ph.D., Koji Igarashi^5^ Ph.D., Kuniyuki Kano^3,6^ Ph.D., Junken Aoki^3,6^ Ph.D., and Makoto Aihara ^1**^ M.D, Ph.D.

1. Department of Ophthalmology, Graduate School of Medicine, The University of Tokyo, Tokyo, Japan

2. Department of Clinical Laboratory Medicine, Graduate School of Medicine, The University of Tokyo, Tokyo, Japan.

3. CREST, Japan Science and Technology Corporation (JST), Saitama, Japan

4. Department of Clinical Laboratory, The University of Tokyo Hospital, Tokyo, Japan

5. Bioscience Division, Reagent Development Department, AIA Research Group, TOSOH Corporation, Kanagawa, Japan

6. Laboratory of Molecular and Cellular Biochemistry, Graduate School of Pharmaceutical Sciences, Tohoku University, Miyagi, Japan

**Supplemental Table S1. Bleb depth, density and vascularization scoring at 1, 3, 6 and 12 months after surgery for each glaucoma subtypes**

| **Variables** | **NTG** | **POAG** | **SOAG** | **XFG** | **P-value‡** |
| --- | --- | --- | --- | --- | --- |
| Bleb depth at 1 month, mean (SD), mm | 0.39 (0.35) | 0.33 (0.19) | 0.16 (0.16) | 0.46 (0.18) | NS |
| [range] | 0-1.123 | 0-0.667 | 0-0.429 | 0.229-0.666 |  |
| Bleb depth at 3 months, mean (SD), mm | 0.29 (0.35) | 0.31 (0.29) | 0.22 (0.19) | 0.31 (0.27) | NS |
| [range] | 0-1.074 | 0-1.125 | 0-0.603 | 0-0.588 |  |
| Bleb depth at 6 months, mean (SD), mm | 0.39 (0.48) | 0.14 (0.23) | 0.34 (0.28) | 0.46 (0.45) | NS |
| [range] | 0-1.49 | 0-0.87 | 0-0.71 | 0-0.895 |  |
| Bleb density at 1 month, mean (SD), optical density unit | 159.4 (16.2) | 132.7 (34.0) | 128.8 (39.0) | 164.2 (22.5) | NS |
| [range] | 140.4-185.39 | 61.637-175.9 | 68.9-157.9 | 134.0-186.3 |  |
| Bleb density at 3 months, mean (SD), optical density unit | 149.8 (32.6) | 136.0 (31.4) | 145.9 (24.1) | 135.0 (46.8) | NS |
| [range] | 93.1-199.8 | 69.7-179.0 | 98.2-180.0 | 63.3-183.6 |  |
| Bleb density at 6 months, mean (SD), mm | 136.0 (37.5) | 132.7 (37.4) | 145.8 (23.8) | 150.1 (23.4) | NS |
| [range] | 74.8-181.0 | 73.7-192.7 | 106.4-169.4 | 123.1-164.8 |  |
| Vascularization at 1 month, mean (SD), grading | 2.4 (1.4) | 2.9 (1.2) | 2.5 (1.3) | 3.0 (0.8) | NS |
| [range] | 1-5 | 1-5 | 1-5 | 2-4 |  |
| Vascularization at 3 months, mean (SD), grading | 2.2 (1.5) | 2.2 (1.1) | 2.3 (1.3) | 3.1 (0.8) | NS |
| [range] | 1-5 | 1-4 | 1-5 | 2-4 |  |
| Vascularization at 6 months, mean (SD), grading | 1.8 (0.9) | 2.4 (1.1) | 2.0 (0.9) | 2.8 (1.0) | NS |
| [range] | 1-3 | 1-4 | 1-4 | 2-4 |  |
| Vascularization at 12 months, mean (SD), grading | 1.7 (0.9) | 2.2 (0.6) | 2.1 (0.5) | 2.0 (0.9) | NS |
| [range] | 1-3 | 1-3 | 1-3 | 1-4 |  |

**Supplemental Table S2. Correlation between bleb depth, density, vascularization scoring and aqueous levels of ATX and LPA at 1, 3, 6 and 12 months after surgery for each glaucoma subtypes**

| **Variables** |  | ATX | LPA |
| --- | --- | --- | --- |
| Bleb depth at 1 month | Spearman’s rank correlation coefficient | 0.048 | -0.08 |
|  | p value | 0.83 | 0.68 |
| Bleb depth at 3 months | Spearman’s rank correlation coefficient | 0.17 | 0.2 |
|  | p value | 0.34 | 0.18 |
| Bleb depth at 6 months | Spearman’s rank correlation coefficient | 0.05 | 0.15 |
|  | p value | 0.8 | 0.35 |
| Bleb density at 1 month | Spearman’s rank correlation coefficient | -0.244 | -0.23 |
|  | p value | 0.272 | 0.23 |
| Bleb density at 3 months | Spearman’s rank correlation coefficient | -0.17 | -0.03 |
|  | p value | 0.32 | 0.84 |
| Bleb density at 6 months | Spearman’s rank correlation coefficient | -0.02 | -0.18 |
|  | p value | 0.92 | 0.25 |
| Vascularization at 1 month | Spearman’s rank correlation coefficient | 0.11 | 0.12 |
|  | p value | 0.48 | 0.39 |
| Vascularization at 3 months | Spearman’s rank correlation coefficient | 0.13 | 0.19 |
|  | p value | 0.44 | 0.18 |
| Vascularization at 6 months | Spearman’s rank correlation coefficient | -0.35 | 0.12 |
|  | p value | 0.83 | 0.39 |
| Vascularization at 12 months | Spearman’s rank correlation coefficient | 0.11 | 0.0009 |
|  | p value | 0.47 | 0.95 |
